# Recent Advances in Comprehending Endothelial Dysfunction and Diabetic Cardiomyopathy: From Molecular Mechanisms to Clinical Applications

**DOI:** 10.3390/jcdd13080377

**Published:** 2026-08-10

**Authors:** Shengying Jia, Li Wang, Zuowei Pei

**Affiliations:** 1 Department of Cardiology, Central Hospital of Dalian University of Technology, Dalian 116033, China; jiasy1983@163.com; 2 Department of Geriatrics, Chengdu Fifth People’s Hospital Affiliated to Chengdu University of Traditional Chinese Medicine, Chengdu 611137, China; 3 Geriatric Diseases Institute of Chengdu, Chengdu 611137, China

**Keywords:** diabetic cardiomyopathy, diabetes mellitus, endothelial dysfunction

## Abstract

Diabetic cardiomyopathy (DCM) is a cardiac condition characterized by various structural and functional abnormalities that are associated with diabetes mellitus. Its development involves multiple factors, among which endothelial dysfunction plays a significant role. To fully describe the relationship between DCM and endothelial dysfunction, we conducted a literature search across several databases, including PubMed, Web of Science, and EMBASE. Our review of DCM research focused on understanding its mechanisms, exploring the methods used to diagnose it, and potential treatment options. Although researchers have made significant progress in DCM diagnosis and treatment in recent years, considerable challenges persist as well. Artificial intelligence (AI)-based multimodal approaches may provide new opportunities for cardiovascular risk stratification and early DCM screening, but DCM-specific models still require external validation before clinical implementation. Regarding treatment options, emerging evidence suggests potential benefits of medications that enhance mitochondrial function and antioxidant properties, as well as anti-inflammatory therapies and lifestyle modifications. Future research should focus on combining different relevant data types, such as genetic and molecular information, to improve the AI tools utilized in medical environments. This study aims to identify novel markers for diagnosing DCM and to devise tailored treatment approaches. These advancements have the potential to improve the prognosis of DCM patients and enhance the identification and management of this condition.

## 1. Introduction

Diabetic cardiomyopathy (DCM) is a medical condition characterized by structural and functional abnormalities in the heart muscle that arise due to diabetes mellitus. Given that DCM bears similarities to coronary artery disease and hypertension [1], it is imperative for medical professionals to diagnose it accurately. DCM commonly presents with myocardial thickening, interstitial fibrosis, concentric remodeling, and diastolic dysfunction; however, it should not be restricted to this phenotype. The ESC recognizes diabetic myocardial disorder as systolic and/or diastolic myocardial dysfunction in the presence of diabetes [2], and diabetes mellitus-related cardiomyopathy has also been described as a dilated phenotype with eccentric left ventricular remodeling and systolic dysfunction. In addition, autoimmune mechanisms may contribute to diabetic cardiomyopathy with systolic dysfunction [3]. Additionally, studies reveal that diabetic patients have a 19–26% chance of developing DCM, with the number of cases steadily expanding [4]. The International Diabetes Federation estimates that by 2030, diabetes mellitus will impact 578 million individuals globally [5], underscoring its significance as a prevalent heart condition. Recent studies have emphasized the role of endothelial dysfunction in DCM progression, which persists as a key factor during all stages of the disease [6].

The connection between endothelial dysfunction and DCM is complex, involving a range of molecular pathways and underlying disease mechanisms. Detecting and treating endothelial dysfunction early currently presents significant challenges to medical practices focused on DCM management, as conventional treatments have not proven particularly successful in arresting DCM advancement. However, treatment approaches that enhance endothelial function have been showing certain promising outcomes [7].

In this review, we searched the PubMed, Web of Science, and EMBASE databases for DCM-related literature published in English between January 2006 and June 2026, with a focus on studies and clinical trials on endothelial dysfunction in terms of its pathogenesis, diagnostic advancements, and treatments undergoing clinical translation. The main goal of this review was to comprehensively compile the current evidence on the subject, outline the latest developments, and suggest potential methods for future research to explore how the management of DCM patients can be further improved.

## 2. Pathophysiology of Endothelial Dysfunction in DCM

Although previous studies have described various mechanisms implicated in the pathogenesis of DCM, such as oxidative stress, inflammation, mitochondrial dysfunction, and fibrosis, the hierarchical relationships among these mechanisms remain ambiguous [7,8]. A key question is whether endothelial dysfunction is merely a downstream consequence of metabolic disturbances in diabetes or an early driving factor in the onset and progression of DCM [7,9]. Accumulating evidence indicates that coronary microvascular endothelial cells are among the early targets of metabolic stress under conditions of chronic hyperglycemia and insulin resistance. Endothelial injury can subsequently reduce nitric oxide (NO) bioavailability, enhance oxidative stress, disrupt the endothelial barrier, and impair paracrine signaling. These changes, in turn, further exacerbate myocardial hypertrophy, fibrosis, and dysfunction [6,7,8,9]. The concept of the “gluco-vascular injury axis,” proposed in recent years, further supports this endothelium-centered research framework. It suggests that diabetes-related metabolic stress can be progressively transmitted to the myocardium via endothelial damage and microvascular dysfunction [10]. Consequently, this paper re-integrates existing research evidence from the perspective of endothelium-driven DCM, with the objective of providing a new mechanistic foundation for the early diagnosis and targeted treatment of DCM [7,9,10].

**Figure 1:**
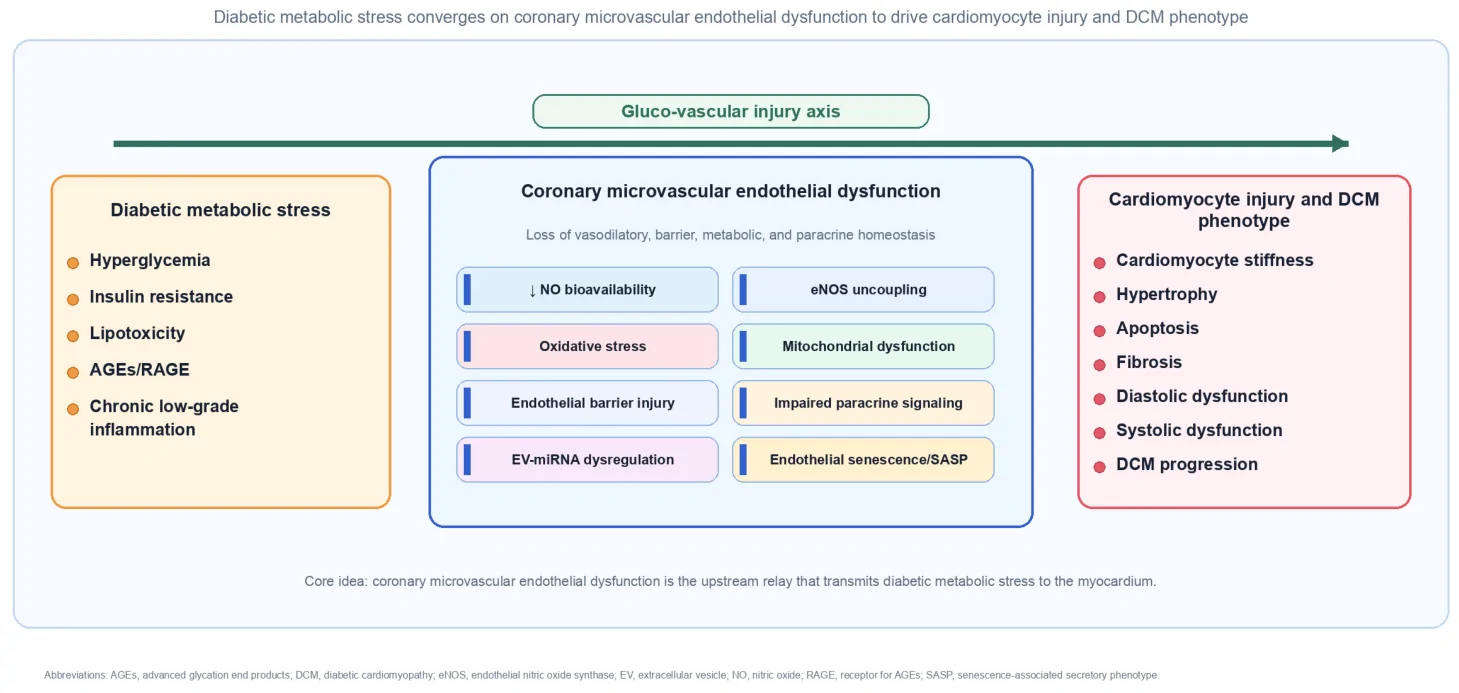
Proposed mechanism linking endothelial dysfunction to diabetic cardiomyopathy (DCM).

As shown in Figure 1, endothelial dysfunction serves as a central upstream event linking metabolic stress to microvascular injury, myocardial remodeling, and the clinical phenotype of DCM.

### 2.1. Molecular Mechanisms Underlying Endothelial Dysfunction

Abnormal endothelial function is crucial during DCM development. Hyperglycemia causes direct harm to endothelial cells through multiple mechanisms, which quickens disease progression. Recent studies have further elucidated the role of circRNA-miRNA-mRNA networks in modulating endothelial dysfunction, providing novel targets for therapeutic intervention [11].

#### 2.1.1. Oxidative Stress Response

Oxidative stress is a central pathological feature of DCM-associated endothelial dysfunction; it is essentially an imbalance between increased reactive oxygen species (ROS) production and insufficient endogenous antioxidant defenses [12,13]. In the diabetic environment, ROS do not originate from a single source but are generated through multiple pathways, including the mitochondrial electron transport chain, nicotinamide adenine dinucleotide phosphate (NADPH) oxidases (NOX), xanthine oxidase (XO), and uncoupled endothelial nitric oxide synthase (eNOS) [12,13]. Among these, electron leakage from mitochondrial complexes I and III promotes the production of superoxide anions; NOX2/NOX4 can amplify oxidative stress in response to stimuli such as hyperglycemia, advanced glycation end products, and angiotensin II; increased XO activity can also further enhance the production of superoxide anions and hydrogen peroxide (H_2_O_2_). These sources of ROS collectively reduce NO bioavailability, impair endothelial barrier function, and promote inflammation, fibrosis, and myocardial microvascular remodeling [12,13].

eNOS uncoupling is a key mechanism in the redox imbalance of the endothelium in diabetes. Under normal conditions, eNOS catalyzes the conversion of L-arginine to NO in a tetrahydrobiopterin (BH4)-dependent manner; however, under oxidative stress in diabetes, BH4 can be oxidized and depleted, causing eNOS to shift from an NO-generating enzyme to a superoxide anion-generating enzyme [12]. Superoxide anions can rapidly react with NO to form peroxynitrite (ONOO^−^), which further reduces the bioavailability of NO and exacerbates damage to endothelial and myocardial cells through mechanisms such as protein nitrosylation, BH4 oxidation, DNA damage, and poly (ADP-ribose) polymerase (PARP) activation [12,14]. This creates a self-amplifying cycle of “increased ROS-decreased NO-ONOO^−^ production-eNOS uncoupling,” which drives diabetes-related endothelial dysfunction and myocardial damage [12,13,14].

ROS can also amplify endothelial damage through various redox-sensitive signaling pathways. The nuclear factor erythroid 2-related factor 2 (Nrf2)/antioxidant response element (ARE) pathway is a critical antioxidant defense system that induces the expression of protective genes such as heme oxygenase-1 (HO-1), NAD(P)H quinone oxidoreductase 1 (NQO1), and glutathione-related enzymes; however, under sustained diabetic stress, this defensive response may be insufficient or dysregulated [12,13]. Concurrently, ROS can activate the nuclear factor-κB (NF-κB) and mitogen-activated protein kinase (MAPK) pathways, promoting the expression of inflammatory and adhesion molecules such as tumor necrosis factor-α (TNF-α), interleukin-1β (IL-1β), interleukin-6 (IL-6), monocyte chemoattractant protein-1 (MCP-1), intercellular adhesion molecule-1 (ICAM-1), and vascular cell adhesion molecule-1 (VCAM-1), thereby enhancing leukocyte adhesion, endothelial inflammation, and microvascular damage [12,13,15]. Abnormalities in redox-sensitive transcription factors such as forkhead box O (FOXO) may also contribute to endothelial cell apoptosis, an imbalance in antioxidant defense, and a decline in vascular repair capacity [12,13]. Therefore, endothelial oxidative stress in diabetes is not merely a simple “increase in ROS and decrease in NO,” but involves multi-source ROS generation, BH4/eNOS uncoupling, ONOO^−^-mediated nitrosation/nitration damage, and abnormalities in redox signaling networks such as Nrf2, NF-κB, MAPK, and FOXO [12,13,14]. Understanding these source-specific ROS pathways and downstream signaling nodes will help in developing more targeted antioxidant intervention strategies, rather than relying solely on nonspecific ROS scavenging [12,13].

#### 2.1.2. The Impact of Insulin Resistance

Insulin resistance not only affects how insulin signaling functions but also reduces endothelial NO production and leads to endothelial dysfunction through various mechanisms. Additionally, it can hinder the phosphoinositide-3 kinase (PI3K)/protein kinase B (Akt) pathway, which lowers NO production and decreases eNOS activity [16]. It can also trigger an increase in endothelin-1 (ET-1) secretion by activating the protein kinase C signaling pathway [17]. As a vasoconstrictor, ET-1 may also exacerbate the various issues associated with vasodilation. Specifically, elevated ET-1 levels can affect blood vessels and their functions within the body [18], and excess levels may result in long-term inflammation, worsened heart muscle fibrosis, and enlarged heart chambers, leading to higher risks of cardiovascular disease progression.

#### 2.1.3. The Binding Between Advanced Glycation End Products (AGEs) and Their Cell Surface Receptors (RAGE)

Under hyperglycemic conditions, glucose and its metabolites react with proteins and other substances to form AGEs, which then bind to RAGE. This triggers both inflammatory reactions and oxidative stress, accelerating vascular remodeling progression and atherosclerosis development [19]. Moreover, the interaction between AGEs and RAGE also worsens the cycle of oxidative stress by increasing ROS production. AGEs amplify the oxidative stress response by activating NADPH oxidase and the mitochondrial oxidative phosphorylation pathway. This interaction induces vascular smooth muscle cells to move, speeding up the formation and advancement of atherosclerotic plaques. They also trigger extracellular matrix degradation by aberrantly activating the matrix metalloproteinase system, weakening the structure and vascular tissue function [20].

#### 2.1.4. Mitochondrial Dysfunction in Diabetic Endothelial Cells

Mitochondrial dysfunction is an important source of oxidative stress and endothelial injury in DCM. Its significance extends beyond impaired adenosine triphosphate (ATP) production and includes disrupted mitochondrial dynamics, defective quality control, impaired biogenesis, and abnormal mitochondria-endoplasmic reticulum (ER) communication [13,21,22].

##### Mitochondrial Fission and Fusion Dynamics

Mitochondria are dynamic organelles that maintain their morphology, distribution, and functional integrity through continuous fission and fusion. Fission is mainly regulated by dynamin-related protein 1 (DRP1) and its outer mitochondrial membrane adaptors, whereas fusion depends on mitofusin 1 (MFN1), mitofusin 2 (MFN2), and optic atrophy 1 (OPA1) [21]. In diabetes-related cardiovascular injury, hyperglycemia, lipotoxicity, and oxidative stress may disturb the fission-fusion balance, promote DRP1-related excessive mitochondrial fission, mitochondrial fragmentation, impaired respiration, and increased ROS generation, thereby aggravating endothelial dysfunction and myocardial microvascular injury [13,21]. Abnormal mitochondrial dynamics may therefore represent an important link between diabetic metabolic stress and endothelial damage.

##### Mitophagy and Mitochondrial Quality Control

Phosphatase and tensin homolog (PTEN)-induced putative kinase 1 (PINK1)/Parkin-mediated mitophagy is a canonical quality-control mechanism for removing damaged mitochondria [21]. When mitochondria become depolarized or injured, PINK1 accumulates on the outer mitochondrial membrane and promotes Parkin recruitment, allowing dysfunctional mitochondria to be selectively eliminated [21]. In diabetes, insufficient mitophagy may lead to the accumulation of fragmented, ROS-producing mitochondria, thereby amplifying oxidative stress, endothelial injury, and cell death signaling [13,21]. In addition to PINK1/Parkin-mediated mitophagy, receptor-mediated pathways such as FUN14 domain containing 1 (FUNDC1)-dependent mitophagy may also participate in mitochondrial quality control, although their endothelial-specific roles in DCM remain insufficiently defined [13,21].

##### Mitochondrial Biosynthesis

Mitochondrial renewal depends not only on the clearance of damaged mitochondria but also on the generation of new mitochondria. Peroxisome proliferator-activated receptor γ coactivator-1α (PGC-1α) is a key transcriptional coactivator that governs mitochondrial biosynthesis and oxidative metabolic capacity; its activity is regulated by energy-sensing signals such as adenosine monophosphate (AMP)-activated protein kinase (AMPK) and sirtuin 1 (SIRT1) [13,21]. In a diabetic environment, hyperglycemia, oxidative stress, and energy metabolism disorders may impair the AMPK-SIRT1-PGC-1α axis, leading to a decline in mitochondrial renewal capacity. When insufficient biosynthesis coexists with defective mitophagy, damaged mitochondria are more likely to accumulate, thereby exacerbating endothelial bioenergetic impairment and ROS production [13,21].

##### Mitochondria-ER Interactions

Mitochondria-associated endoplasmic reticulum membranes (MAMs) serve as contact zones between mitochondria and the endoplasmic reticulum that regulate calcium (Ca^2+^) transport, lipid metabolism, mitochondrial dynamics, autophagosome formation, apoptosis, and the regulation of inflammatory signaling [22]. In diabetic microvascular complications, MAM abnormalities are believed to be associated with diabetic kidney disease, DCM, and diabetic retinopathy [22]. Dysfunction of MAMs may disrupt mitochondria-ER Ca^2+^ homeostasis, enhance mitochondrial dysfunction, ER stress, and cell death signaling, thereby further disrupting endothelial mitochondrial homeostasis and promoting diabetes-related microvascular damage [22].

##### Therapeutic Implications

Endothelial mitochondrial damage in diabetes should be understood as an integrated disturbance involving mitochondrial dynamics, quality control, biogenesis, and organelle communication rather than ATP deficiency alone [13,21,22]. Targeting mitochondrial homeostasis has potential therapeutic significance; however, interventions that directly enhance mitochondrial quality control or mitochondrial protection remain primarily in the research phase for DCM. Previous studies have shown that dapagliflozin can improve endothelial mitochondrial respiration independently of its hypoglycemic effects, suggesting that some of its cardiovascular protective effects may be related to endothelial mitochondrial mechanisms [23].

### 2.2. The Role of Endothelial Dysfunction in Myocardial Injury

Endothelial dysfunction affects heart muscle health through multiple complex mechanisms that disturb the metabolic equilibrium and energy supply.

#### 2.2.1. The Deterioration of Endothelial Cell Function

Endothelial dysfunction reduces NO bioavailability, impairs microcirculation, and limits myocardial perfusion. As a result, mitochondrial oxidative phosphorylation becomes less efficient, and glycolytic compensation is altered, leading to myocardial energy imbalance and metabolic dysfunction.

#### 2.2.2. Endothelial Cell-Cardiomyocyte Paracrine and Metabolic Communication

The transmission of endothelial dysfunction to myocardial cell damage is not a single process but is jointly mediated through paracrine signaling, extracellular vesicles (EVs), and metabolic coupling [7,8,9]. Under physiological conditions, coronary microvascular endothelial cells maintain homeostasis by releasing vasoactive mediators; however, in the presence of chronic hyperglycemia and insulin resistance, protective endothelial signaling is attenuated while damaging signaling is enhanced, which can promote myocardial metabolic dysfunction, inflammation, fibrosis, and dysfunction [7,8,10]. The NO-soluble guanylate cyclase (sGC)-cyclic guanosine monophosphate (cGMP)-protein kinase G (PKG) pathway is a crucial paracrine axis linking endothelial function to myocardial diastolic function. In diabetes, decreased eNOS activity, eNOS uncoupling, and increased oxidative stress decrease NO bioavailability, thereby weakening cGMP-PKG signaling in cardiomyocytes. This may increase cardiomyocyte passive stiffness, hypertrophy, and fibrosis, and ultimately exacerbate diastolic dysfunction [7,8,12,13]. Conversely, vasoconstrictive and pro-remodeling mediators such as ET-1 may be relatively enhanced during diabetic endothelial dysfunction and contribute to myocardial remodeling through inflammatory, oxidative, and profibrotic signaling [8,17,18]. Beyond classical vasoactive mediators, endothelial-derived protective growth factors also participate in endothelial-cardiomyocyte communication. Neuregulin-1 (NRG1) can be secreted by cardiovascular endothelial cells and act on ErbB receptor tyrosine kinase 2/4 (ErbB2/ErbB4) on neighboring cardiomyocytes, thereby activating downstream protective pathways and helping maintain cardiomyocyte function under diabetic stress [24,25]. In addition, endothelial-derived EVs can carry microRNAs (miRNAs), proteins, and lipids that transmit metabolic and inflammatory signals to cardiomyocytes. Recent evidence indicates that circulating exosomal miR-550a-5p and miR-665 are associated with coronary microvascular dysfunction and myocardial injury markers in patients with type 2 diabetes, suggesting that EV-mediated miRNA transfer may participate in the propagation of coronary microvascular injury to cardiomyocyte damage [26,27].

Endothelial cells may also regulate myocardial energy metabolism by controlling the transendothelial transport of substrates such as glucose and fatty acids [8]. In diabetes, endothelial insulin resistance and altered substrate transport may shift myocardial substrate utilization toward greater fatty acid dependence, thereby aggravating lipid accumulation, mitochondrial dysfunction, oxidative stress, and lipotoxic injury [8,13]. Thus, endothelial dysfunction in DCM should not be considered a passive vascular abnormality. Rather, dysfunctional endothelial cells may transmit diabetes-related metabolic stress to cardiomyocytes through NO/ET-1 imbalance, impaired NRG1-ErbB protective signaling, EV-miRNA transfer, and disrupted metabolic coupling, thereby contributing to cardiomyocyte hypertrophy, fibrosis, apoptosis, and systolic/diastolic dysfunction [7,8,9,10,24,25,26,27].

#### 2.2.3. Inflammatory Activation and Immune-Endothelial Interactions

Inflammatory activation and immune-endothelial interactions may further amplify endothelial dysfunction and myocardial microvascular injury in DCM.

##### NLRP3 Inflammasome Activation

The NLR family pyrin domain containing 3 (NLRP3) inflammasome is an important signaling platform linking diabetic metabolic stress to sterile inflammation [28]. Under stimuli such as hyperglycemia, lipid overload, and increased mitochondrial ROS, NLRP3 can be activated and promote caspase-1-mediated maturation of IL-1β and IL-18 as well as gasdermin D (GSDMD)-dependent pyroptosis, thereby amplifying endothelial inflammation and myocardial microvascular injury [28]. Recent studies also suggest that mitochondrial damage and mitochondrial DNA (mtDNA) leakage may activate the cyclic GMP-AMP synthase (cGAS)-stimulator of interferon genes (STING) pathway, which further promotes diabetes-related cardiovascular inflammation and pyroptosis through NF-κB/NLRP3-related signaling [28]. Thus, NLRP3 and its upstream cGAS-STING axis may represent important nodes of inflammatory amplification in DCM [28].

##### Immunometabolism and Endothelial Metabolic Reprogramming

Hyperglycemia, lipid overload, and oxidative stress in diabetes can alter the metabolic states of vascular and immune cells and promote chronic low-grade inflammation [13,29]. Immunometabolic studies suggest that enhanced glycolysis, mitochondrial dysfunction, and abnormal metabolic sensing through pathways such as hypoxia-inducible factor-1α (HIF-1α), AMPK, and mammalian target of rapamycin (mTOR) may promote inflammatory phenotypes and cytokine release [29]. In addition, persistent metabolic stress may induce metabolic and epigenetic reprogramming that maintains heightened proinflammatory responsiveness in monocytes/macrophages, contributing to residual inflammatory risk and cardiovascular remodeling; however, direct evidence for this trained immunity concept in DCM remains limited [29]. Therefore, endothelial metabolic abnormalities and immunometabolic imbalance may jointly contribute to microvascular inflammation and myocardial remodeling in DCM, although cell-specific mechanisms require further evidence [13,29].

##### Macrophage Polarization and the Cardiac Immune Microenvironment

Macrophage-mediated inflammation may contribute to diabetes-related myocardial injury, endothelial activation, and tissue remodeling [29]. Under hyperglycemic and lipotoxic stress, macrophage metabolic reprogramming may favor proinflammatory phenotypes and increase the release of inflammatory mediators such as TNF-α, IL-1β, and IL-6 [29]. These signals can amplify local inflammation and promote endothelial dysfunction, cardiomyocyte injury, and fibrotic remodeling; however, specific macrophage subsets in the diabetic heart and their relationship with endothelial-to-mesenchymal transition (EndMT) remain to be further clarified [29]. Thus, macrophage polarization is better considered a potential regulatory component of the inflammatory microenvironment in DCM rather than an established disease-specific therapeutic target [29].

##### Endothelial Cell Senescence and SASP

Hyperglycemia, oxidative stress, and mitochondrial dysfunction may promote endothelial injury and induce cellular senescence and a senescence-associated secretory phenotype (SASP) [30]. Senescent endothelial cells can release proinflammatory and profibrotic mediators, thereby amplifying local inflammation, endothelial dysfunction, and tissue remodeling through paracrine mechanisms [30,31]. However, the causal role of endothelial senescence in coronary microvascular injury during DCM remains incompletely defined, and senescent cell clearance strategies should currently be regarded as a potential research direction [31].

Overall, inflammation in DCM is not limited to elevated classical cytokines. It involves multiple layers, including NLRP3/cGAS-STING-mediated inflammasome activation, immunometabolic imbalance, macrophage inflammatory polarization, endothelial cell senescence/SASP, and reciprocal interactions between immune cells and endothelial cells [28,29,30,31]. These interconnected processes may jointly sustain diabetes-related microvascular inflammation, endothelial dysfunction, and myocardial remodeling, but their causal roles and therapeutic targetability in DCM require further validation.

**Figure 2:**
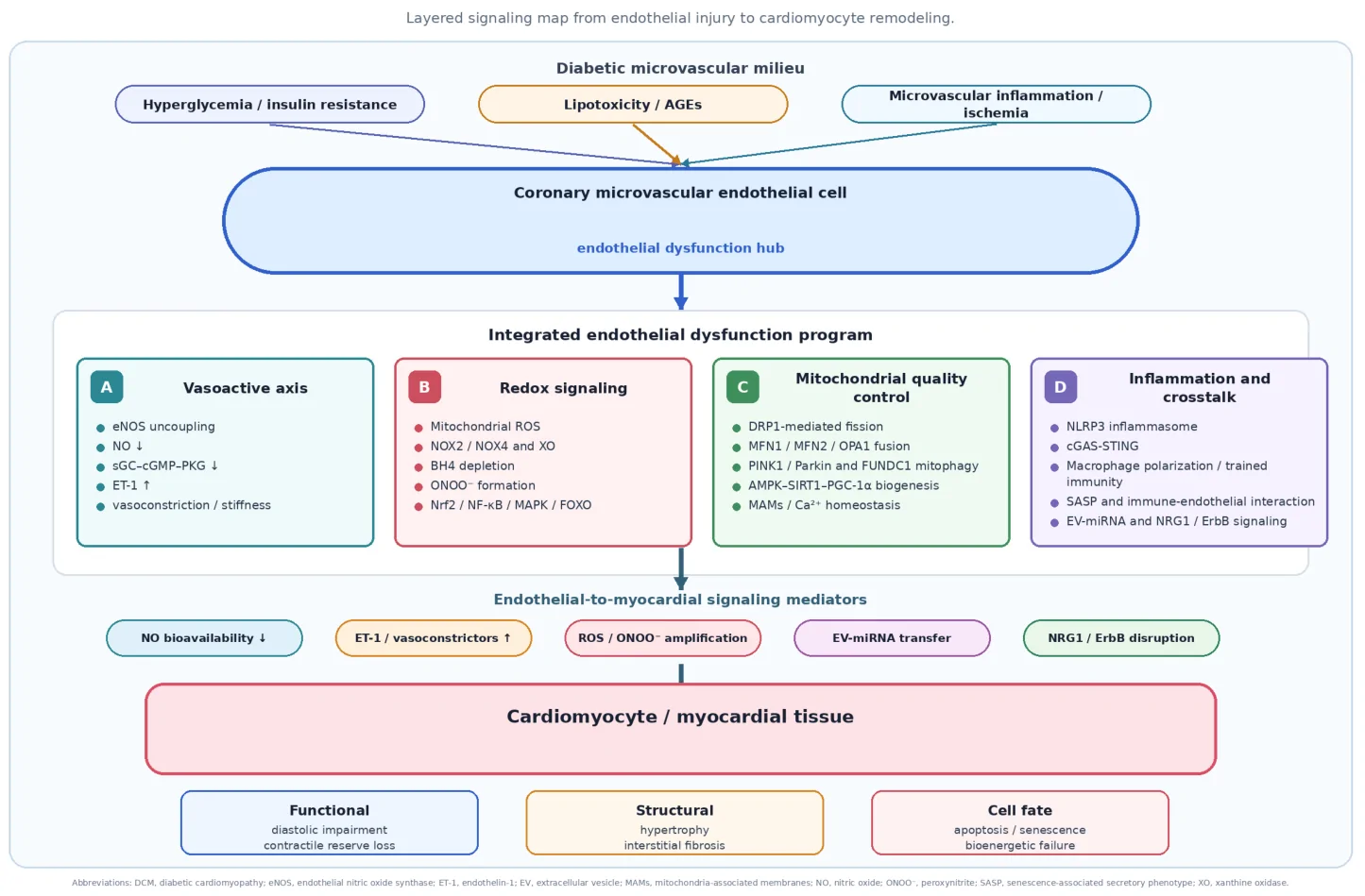
Molecular mechanisms linking endothelial dysfunction to myocardial injury in diabetic cardiomyopathy. Abbreviations: AGE, Advanced glycation end product; eNOS, Endothelial nitric oxide synthase; NO, Nitric oxide; sGC, Soluble guanylate cyclase; cGMP, Cyclic guanosine monophosphate; PKG, Protein kinase G; ET-1, Endothelin-1; ROS, Reactive oxygen species; NOX, Nicotinamide adenine dinucleotide phosphate (NADPH) oxidases; BH4, Tetrahydrobiopterin; ONOO^−^, Peroxynitrite; Nrf2, The nuclear factor erythroid 2-related factor 2; NF-κB, Nuclear factor-κB; MAPK, Mitogen-activated protein kinase; FOXO, Forkhead box O; DRP1, Dynamin-related protein 1; MFN1, Mitofusin 1; OPA1, Optic atrophy 1; PINK1, PTEN-induced putative kinase 1; FUNDC1, FUN14 domain containing 1; AMPK, Adenosine monophosphate (AMP)-activated protein kinase; SIRT1, Sirtuin 1; PGC-1α, Peroxisome proliferator-activated receptor γ coactivator-1α; MAMs, Mitochondria-associated endoplasmic reticulum membranes; Ca^2+^;, Calcium; NLRP3, Nucleotide-binding oligomerization domain-like receptor family, pyrin domain containing 3; cGAS, Cyclic GMP-AMP synthase; STING, (cGAS)-stimulator of interferon genes; SASP, Senescence-associated secretory phenotype; EVs, Extracellular vesicles; MiRNAs, microRNAs; NRG1, Neuregulin-1; ErbB2/ErbB4, ErbB receptor tyrosine kinase 2/4. Diabetic metabolic stress promotes coronary microvascular endothelial dysfunction through impaired NO-sGC-cGMP-PKG signaling, ET-1 activation, oxidative and nitrosative stress, mitochondrial quality-control defects, inflammatory activation, EV-miRNA transfer, and impaired NRG1/ErbB signaling. These mechanisms may collectively contribute to myocardial stiffness, hypertrophy, fibrosis, apoptosis, bioenergetic failure, diastolic impairment, and reduced contractile reserve.

The mechanisms described above are summarized in Figure 2, which illustrates how endothelial dysfunction may promote myocardial injury through impaired signaling, oxidative stress, inflammation, mitochondrial dysfunction, and disrupted endothelial-cardiomyocyte communication.

### 2.3. The Role of Endothelial Dysfunction in Ventricular Remodeling

Ventricular remodeling represents the most common pathological alteration in DCM. It is characterized by myocardial fibrosis, dysregulated extracellular matrix remodeling, and an enlarged ventricular cavity. This pathological progression is further enhanced by endothelial dysfunction through a variety of pathophysiological mechanisms.

#### 2.3.1. Alterations in Vascular Permeability

Endothelial dysfunction increases the movement of inflammatory cells into heart tissue. This ongoing inflammatory response accelerates myocardial fibrosis development and the structural organization of the extracellular matrix, both of which disrupt the homeostasis of the myocardial microenvironment [32].

#### 2.3.2. Apoptosis and Necrosis

Endothelial dysfunction leads to both programmed cell death (i.e., apoptosis) and uncontrolled cell death (i.e., necrosis) in cardiomyocytes by initiating oxidative stress and activating various inflammatory responses [14]. As the number of cardiomyocytes decreases, the ventricular wall gradually loses its elasticity, which directly reduces its flexibility and causes a decline in both systolic and diastolic functions.

#### 2.3.3. Microvascular Lesions and Myocardial Ischemia

A close connection exists between microvascular lesions and myocardial ischemia. Research has demonstrated a relationship between endothelial dysfunction and microvascular lesions in the heart muscle, which may lead to reduced blood flow to the heart and disturb the equilibrium of metabolic processes [33]. Additionally, microcirculation disturbances decrease cardiomyocyte oxygen levels and nutrient uptake efficiency while concurrently driving the processes involved in pathological ventricular remodeling.

DCM progression is facilitated by endothelial dysfunction through multiple pathways, including oxidative stress and inflammatory responses, while disease complexity involves metabolic disorders and mitochondrial dysfunction. Thus, understanding these mechanisms comprehensively may foster a better understanding of DCM that facilitates the development of preventative strategies and targeted therapeutic approaches.

## 3. Diagnosis

Endothelial dysfunction, the central pathological mechanism underlying DCM, ranges from microcirculatory abnormalities to structural remodeling. In clinical practice, physicians must properly ascertain and evaluate endothelial function. However, owing to the low specificity of traditional detection methods, it is difficult to identify DCM during its early stages. Therefore, researchers urgently need to develop better detection technologies to identify the pathological characteristics associated with this condition.

### 3.1. Candidate Biomarkers of Endothelial Dysfunction in DCM: Current Evidence and Limitations

Cardiac fatty acid-binding protein 3 (FABP3) is highly expressed in cardiomyocytes and can be released into the circulation after myocardial injury. In ambulatory patients with chronic heart failure and type 2 diabetes, higher FABP3 levels independently predicted all-cause and cardiovascular mortality [34]. However, FABP3 elevation is not specific to DCM and may also occur in acute coronary syndrome, various cardiomyopathies, and heart failure. Current evidence therefore supports FABP3 more as a marker of myocardial injury and prognostic risk stratification than as a DCM-specific diagnostic biomarker [34]. Similarly, insulin-like growth factor-binding protein 7 (IGFBP7) is associated with heart failure, aging, metabolic disturbance, and fibrosis and has shown risk-stratification value in both heart failure with reduced ejection fraction (HFrEF) and heart failure with preserved ejection fraction (HFpEF). Nevertheless, IGFBP7 reflects a broader heart failure and cardiorenal-metabolic context rather than a DCM-specific pathological process [35]. Matrix metalloproteinase-9 (MMP-9) primarily reflects extracellular matrix degradation, inflammation, and myocardial remodeling. Because MMP-9 can be elevated in heart failure, dilated cardiomyopathy, coronary artery disease, and hypertension-related cardiac remodeling, it is better considered a remodeling-related candidate biomarker rather than a DCM-specific marker [36].

Circulating non-coding RNAs (ncRNAs), particularly exosomal miRNAs, may more closely reflect early microvascular and endothelial injury in DCM [37,38]. Recent evidence indicates that circulating exosomal miR-550a-5p and miR-665 are associated with coronary microvascular dysfunction and myocardial injury risk in patients with type 2 diabetes, suggesting potential value for early detection and risk stratification [26]. However, exosomal miRNA measurement remains affected by variability in EV isolation, normalization, and reporting workflows. Moreover, current evidence is mainly derived from single-center, cross-sectional, or retrospective studies, and the incremental value of these markers beyond conventional biomarkers such as cardiac troponin I (cTnI) and N-terminal pro-B-type natriuretic peptide (NT-proBNP) requires validation in prospective multicenter cohorts [26].

Overall, no single standardized biomarker is currently sufficiently specific for routine DCM diagnosis. A more feasible future approach may involve integrating microvascular/endothelial markers, myocardial injury or heart failure-related biomarkers, and metabolic indicators into multidimensional risk-stratification models, although this strategy requires validation in prospective, multicenter human cohorts [26,34,38].

These candidate biomarkers and their main strengths and limitations are summarized in Table 1.

### 3.2. Artificial Intelligence in DCM Diagnosis: Current Opportunities and Limitations

Artificial intelligence (AI) provides new opportunities for cardiovascular screening, risk stratification, and prognostication. Current evidence suggests that AI can integrate electrocardiography, cardiac imaging, electronic health records, wearable-device signals, circulating biomarkers, and multi-omics data to identify digital biomarkers, screen for structural heart disease risk, and predict clinical outcomes [39]. In the context of DCM, such multimodal approaches may help detect early myocardial remodeling, fibrosis, edema, microvascular dysfunction, and endothelial injury-related signatures [7,39]. However, the application of AI in DCM remains at an early stage. Adequately externally validated and real-world-tested DCM-specific AI models are still lacking. Before clinical implementation, such tools require further evaluation of diagnostic performance, generalizability, interpretability, data security, algorithmic fairness, workflow interoperability, and regulatory feasibility [39].

### 3.3. Future Development Direction

#### 3.3.1. Integrative Analysis of Multi-Omics Data

Several novel biomarkers and molecular signals associated with endothelial dysfunction have recently been explored in DCM-related research. However, their diagnostic specificity, sensitivity, and incremental value beyond conventional markers remain insufficiently established. Future research should integrate metabolomics, proteomics, transcriptomics, imaging features, and clinical variables to develop multidimensional risk-stratification models for early DCM assessment and individualized monitoring. These approaches require validation in prospective, multicenter cohorts before clinical application.

#### 3.3.2. Optimization of Non-Invasive Detection Technology

Such efforts may benefit from concentrating on the advancement of non-invasive, highly accurate, and effective techniques for assessing endothelial function. More precisely, flow-mediated dilation (FMD) detection technology simplifies operational complexities and minimizes the possibility of operational error. Broader clinical adoption of these techniques will require further validation and standardization.

## 4. Treatment

Current management of DCM remains based on glycemic control, cardiovascular risk-factor management, lifestyle intervention, and standard heart failure therapy when clinically indicated. However, because endothelial dysfunction is increasingly recognized as an important contributor to DCM progression, therapies with potential endothelial protective effects have attracted increasing attention.

### 4.1. Endothelial Protective Effects of Contemporary Glucose-Lowering Therapies

#### 4.1.1. SGLT2 Inhibitors: From Glucose Excretion to Endothelial Repair

In recent years, the cardiovascular benefits of sodium-glucose cotransporter 2 (SGLT2) inhibitors have attracted substantial attention, and some of these effects may not be entirely dependent on glucose lowering [40]. At the endothelial level, SGLT2 inhibitors may exert protective effects by reducing oxidative stress, improving AMPK/eNOS-related NO bioavailability, modulating energy-sensing pathways such as AMPK/mTOR, and attenuating inflammatory activation [40]. Notably, dapagliflozin has been shown to directly improve endothelial mitochondrial respiration, relatively independently of its glucose-lowering effect [23]. These pleiotropic mechanisms may partly explain the cardiovascular benefits observed with SGLT2 inhibitors.

#### 4.1.2. GLP-1 Receptor Agonists: Endothelial Protection Beyond Incretin Effects

Glucagon-like peptide-1 receptor agonists (GLP-1 RAs) have demonstrated cardiovascular benefit in multiple large cardiovascular outcome trials enrolling patients with type 2 diabetes and high cardiovascular risk [41]. Beyond glucose lowering and weight reduction, the vascular protective effects of GLP-1 RAs may be partly related to improved endothelial function [41]. Current evidence suggests that potential mechanisms include attenuation of oxidative and endoplasmic reticulum stress, improvement of mitochondrial function, enhancement of eNOS-dependent NO bioavailability, improvement of endothelium-dependent vasodilation, and reduction in inflammatory activation and adhesion molecule expression such as ICAM-1 and VCAM-1 [41]. In the context of DCM, GLP-1 RAs should be regarded as systemic cardiometabolic therapies with potential indirect endothelial protective effects rather than established DCM-specific endothelial-targeted treatments. Direct evidence that GLP-1 RAs restore endothelial-cardiomyocyte communication or reverse coronary microvascular endothelial dysfunction in DCM remains limited [41].

### 4.2. Emerging Therapeutic Strategies Targeting Endothelial Dysfunction and DCM

#### 4.2.1. Mitochondria-Targeted Therapy

Mitochondrial dysfunction is an important component of endothelial injury and myocardial energy metabolic disturbance in DCM; therefore, mitochondria-targeted therapy has been considered a potential intervention strategy. MitoQ (mitoquinone) is a mitochondria-targeted ubiquinone derivative, and preclinical as well as early human studies suggest that it may improve vascular endothelial function. In hypertensive rats, MitoQ10 increased aortic NO bioavailability, improved endothelial function, and attenuated cardiac hypertrophy [42]. In healthy older adults, 6 weeks of MitoQ supplementation improved brachial artery flow-mediated dilation and reduced mitochondrial ROS-related suppression of endothelial function [43]. In addition, elamipretide/SS-31 is a peptide compound that targets inner mitochondrial membrane cardiolipin and may restore mitochondrial bioenergetics by stabilizing cardiolipin-cytochrome C interactions, preserving cristae structure, and improving oxidative phosphorylation; animal heart failure studies suggest potential benefits for myocardial function and remodeling [44]. However, direct clinical evidence for MitoQ and elamipretide/SS-31 in patients with DCM or HFpEF remains limited; therefore, these approaches should currently be regarded as mechanistically plausible but exploratory strategies rather than established DCM therapies [42,43,44].

#### 4.2.2. Senolytic Therapy

Endothelial cell senescence and the SASP may link diabetes-related metabolic stress, chronic low-grade inflammation, oxidative stress, and cardiovascular injury [30]. In this context, senolytic strategies targeting senescent cells, such as dasatinib plus quercetin, fisetin, and agents targeting anti-apoptotic proteins of the B-cell lymphoma 2 (BCL-2) family, have emerged as potential directions in cardiovascular translational research [31]. However, current evidence is mainly derived from animal studies and indirect cardiovascular research. Their efficacy, safety in DCM, and their ability to improve coronary microvascular endothelial function remain unvalidated.

#### 4.2.3. RNA-Based Therapeutics

ncRNAs may participate in DCM progression by regulating programmed cell death processes, including apoptosis, autophagy, pyroptosis, ferroptosis, and necroptosis, and are therefore considered potential mechanistic and interventional targets [37]. Existing studies suggest that modulation of specific ncRNAs may influence DCM-related cell death and myocardial injury. However, the available evidence is mainly derived from animal models and cellular experiments, and cell type-specific delivery, long-term safety, off-target effects, and clinical translatability require further validation [37].

#### 4.2.4. Stem Cell-Related Therapeutic Strategies

Stem cell-related therapeutic strategies have also been explored as potential interventions for DCM. Previous reviews suggest that mesenchymal stem cell therapy and stem cell transplantation may show cardioprotective potential in DCM models [39]. However, these approaches still face challenges related to cell source, manufacturing standardization, in vivo survival, long-term safety, and clinical translatability. Therefore, they should currently be regarded as investigational therapeutic directions [38].

## 5. Conclusions

The review provided an in-depth analysis of the link between DCM and endothelial dysfunction. Endothelial dysfunction plays a key role in DCM progression by affecting myocardial structure and function through metabolic dysregulation, oxidative stress, inflammation, mitochondrial irregularities, impaired paracrine signaling, and microvascular injury. Recent technological developments, including magnetic resonance imaging (MRI) and AI-based approaches, may provide new opportunities for assessing endothelial dysfunction and improving risk stratification in DCM, although DCM-specific AI models still require further external validation before routine clinical application.

In the clinical management of DCM, traditional glucose-lowering medications remain a cornerstone, but newer therapeutic approaches, including SGLT2 inhibitors and GLP-1 receptor agonists and targeted endothelial-protective strategies, may help improve endothelial function. Future research in this field should prioritize comprehensive analyses of multi-omics data, the validation of multimodal risk-stratification models, and the formulation of personalized treatment plans for patients. By integrating these elements, future studies may improve the early detection, risk assessment, and personalized management of DCM. Whether these strategies can improve patient prognosis and clinical outcomes requires further validation in prospective studies.

## Figures and Tables

**Table 1:** Representative Biomarkers in Diabetic Cardiomyopathy: Biological Signals, Clinical Evidence, and Limitations.

Biomarker	Biological Source/Pathological Signal	Clinical Evidence/Potential Value in DCM	Major Limitations
Cardiac fatty acid-binding protein 3 (FABP3)	Mainly expressed in cardiomyocytes; reflects myocardial injury and disturbed fatty acid metabolism.	In chronic heart failure patients with type 2 diabetes, higher FABP3 levels independently predicted all-cause and cardiovascular mortality [20].	Not specific to DCM. Robust diagnostic sensitivity and specificity for DCM have not been established [20].
Insulin-like growth factor-binding protein 7 (IGFBP7)	Associated with aging, fibrosis, metabolic stress, endothelial dysfunction, and heart failure-related remodeling.	Has shown risk-stratification value in heart failure phenotypes, including heart failure with HFrEF and HFpEF [22].	Reflects a broad heart failure and cardiorenal-metabolic context rather than a DCM-specific process [22].
Matrix metalloproteinase-9 (MMP-9)	Reflects extracellular matrix degradation, inflammation, and myocardial remodeling/fibrosis.	May serve as a candidate marker of myocardial remodeling and fibrotic activity in DCM-related structural injury [23].	Elevated in multiple cardiovascular diseases, including coronary artery disease, dilated cardiomyopathy, hypertension-related remodeling, and heart failure [23].
Exosomal miR-550a-5p/miR-665	Extracellular vesicle (EV)-derived microRNAs linked to coronary microvascular dysfunction and endothelial-myocardial injury.	Circulating exosomal miR-550a-5p and miR-665 are associated with coronary microvascular dysfunction and myocardial injury in type 2 diabetes and may help early risk stratification [25].	Evidence is mainly from single-center, cross-sectional, or retrospective studies. EV isolation, normalization, and reporting workflows remain non-standardized [25].
Circulating non-coding RNAs (ncRNAs)	Related to programmed cell death, fibrosis, inflammation, mitochondrial dysfunction, and endothelial injury.	May provide mechanistic and diagnostic information because ncRNAs participate in DCM-related apoptosis, autophagy, pyroptosis, ferroptosis, and necroptosis [24,28].	Assay standardization, reproducibility, disease specificity, and clinical interpretability remain limited, and clinically validated DCM cutoffs and performance metrics are lacking [24,28].

Abbreviations: DCM, diabetic cardiomyopathy; FABP3, fatty acid-binding protein 3; IGFBP7, insulin-like growth factor-binding protein 7; MMP-9, matrix metalloproteinase-9; EV, extracellular vesicle; ncRNA, non-coding RNA; cTn, cardiac troponin; NT-proBNP, N-terminal pro-B-type natriuretic peptide; HFrEF, heart failure with reduced ejection fraction; HFpEF, heart failure with preserved ejection fraction; TIMPs, tissue inhibitors of metalloproteinases.

## Data Availability

No new data were created or analyzed in this study. Data sharing is not applicable to this article.

## Abbreviations

The following abbreviations are used in this manuscript:

DCM	Diabetic cardiomyopathy
AI	Artificial intelligence
NO	Nitric oxide
ROS	Reactive oxygen species
NADPH	Nicotinamide adenine dinucleotide phosphate
NOX	NADPH oxidases
XO	Xanthine oxidase
eNOS	Endothelial nitric oxide synthase
H2O2	Hydrogen peroxide
BH4	Tetrahydrobiopterin
ONOO^−^	Peroxynitrite
PARP	Poly (ADP-ribose) polymerase
Nrf2	The nuclear factor erythroid 2-related factor 2
ARE	Antioxidant response element
HO-1	Heme oxygenase-1
NQO1	NAD(P)H quinone oxidoreductase 1
NF-κB	Nuclear factor kappa B
MAPK	Mitogen-activated protein kinase
TNF-α	Tumor necrosis factor-α
IL	Interleukin
MCP-1	Monocyte chemoattractant protein-1
ICAM-1	Intercellular adhesion molecule-1
VCAM-1	Vascular cell adhesion molecule-1
FOXO	Forkhead box O
PI3K	Phosphatidylinositol-3 kinase
Akt	Protein kinase B
ET-1	Endothelin-1
AGE	Advanced glycation end product
RAGE	Receptor for advanced glycation end product
ATP	Adenosine triphosphate
ER	Endoplasmic reticulum
DRP1	Dynamin-related protein 1
MFN	Mitofusin
OPA1	Optic atrophy 1
PTEN	Phosphatase and tensin homolog
PINK1	PTEN-induced putative kinase 1
FUNDC1	FUN14 domain containing 1
PGC-1α	Peroxisome proliferator-activated receptor γ coactivator-1α
AMP	Adenosine monophosphate
AMPK	AMP-activated protein kinase
SIRT1	Sirtuin 1
MAMs	Mitochondria-associated endoplasmic reticulum membranes
Ca^2+^	Calcium
EVs	Extracellular vesicles
sGC	Soluble guanylate cyclase
cGMP	Cyclic guanosine monophosphate
PKG	Protein kinase G
NRG1	Neuregulin-1
ErbB2/ErbB4	ErbB receptor tyrosine kinase 2/4
miRNA	microRNAs
NLRP3	Nucleotide-binding oligomerization domain-like receptor family, pyrin domain containing 3
GSDMD	Gasdermin D
mtDNA	Mitochondrial DNA
cGAS	Cyclic GMP-AMP synthase
STING	(cGAS)-stimulator of interferon genes
HIF-1α	Hypoxia-inducible factor-1α
mTOR	Mammalian target of rapamycin
EndMT	Endothelial-to-mesenchymal transition
SASP	Senescence-associated secretory phenotype
FABP3	Fatty acid binding protein 3
IGFBP7	Insulin-like growth factor binding protein 7
HFrEF	Heart failure with reduced ejection fraction
HFpEF	Heart failure with preserved ejection fraction
MMP	Matrix metalloproteinase
ncRNAs	Non-coding RNAs
cTnI	Cardiac troponin I
NT-proBNP	N-terminal pro-B-type natriuretic peptide
FMD	Flow-mediated dilation
SGLT2	Sodium–glucose transport protein 2
GLP-1	Glucagon-like peptide-1
BCL-2	B-cell lymphoma 2
MRI	Magnetic resonance imaging
